# Outcome Measures in Facioscapulohumeral Muscular Dystrophy Clinical Trials

**DOI:** 10.3390/cells11040687

**Published:** 2022-02-16

**Authors:** Mehdi Ghasemi, Charles P. Emerson, Lawrence J. Hayward

**Affiliations:** 1 Department of Neurology, University of Massachusetts Chan Medical School, Worcester, MA 01655, USA; charles.emersonjr@umassmed.edu (C.P.E.J.); lawrence.hayward@umassmed.edu (L.J.H.); 2 Wellstone Muscular Dystrophy Program, Department of Neurology, University of Massachusetts Chan Medical School, Worcester, MA 01655, USA

**Keywords:** facioscapulohumeral muscular dystrophy (FSHD), double homeobox 4 (*DUX4*), clinical trial, outcome measures, magnetic resonance imaging (MRI)

## Abstract

Facioscapulohumeral muscular dystrophy (FSHD) is a debilitating muscular dystrophy with a variable age of onset, severity, and progression. While there is still no cure for this disease, progress towards FSHD therapies has accelerated since the underlying mechanism of epigenetic derepression of the double homeobox 4 (*DUX4*) gene leading to skeletal muscle toxicity was identified. This has facilitated the rapid development of novel therapies to target *DUX4* expression and downstream dysregulation that cause muscle degeneration. These discoveries and pre-clinical translational studies have opened new avenues for therapies that await evaluation in clinical trials. As the field anticipates more FSHD trials, the need has grown for more reliable and quantifiable outcome measures of muscle function, both for early phase and phase II and III trials. Advanced tools that facilitate longitudinal clinical assessment will greatly improve the potential of trials to identify therapeutics that successfully ameliorate disease progression or permit muscle functional recovery. Here, we discuss current and emerging FSHD outcome measures and the challenges that investigators may experience in applying such measures to FSHD clinical trial design and implementation.

## 1. Introduction

**Figure 1:**
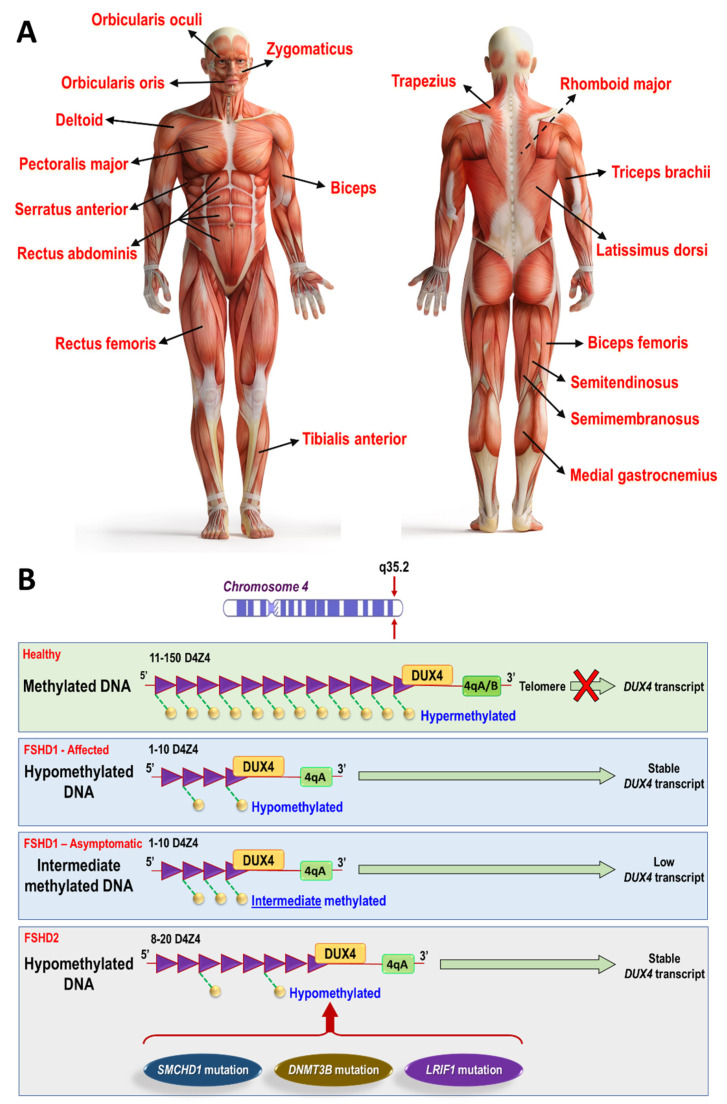
(**A**) FSHD preferentially involves muscles of the face, shoulder girdle, upper arm, and some leg muscles. The pattern of weakness is typically asymmetrical and quite variable in onset, severity, and progression. Facial weakness can be subtle. Some arm muscles such as deltoid may be only partially affected compared to biceps and triceps, and some leg muscles such as tibialis anterior can be affected at early stages. (**B**) Genetic and epigenetic abnormalities underlying FSHD. In healthy individuals, the D4Z4 retrotransposon repeat array near the telomere of chromosome 4q contains 11 to 150 highly methylated 3.3 kb repeats and effectively silences the *DUX4* gene. In affected FSHD1 patients, contraction of the locus to 1–10 repeat units is associated with local DNA hypomethylation and in the presence of a permissive 4qA allele allows *DUX4* to produce a stable polyadenylated transcript. In non-manifesting or unaffected FSHD1 individuals, there are also few D4Z4 repeats, but these have higher methylation (i.e., intermediate methylation) compared to affected or symptomatic FSHD1 patients. In FSHD2, contraction of the locus to 8–20 repeat units in the context of the 4qA haplotype plus an additional mutation in chromatin modifier genes such as *SMCHD1* (structural maintenance of chromosomes flexible hinge domain containing 1) produces even greater D4Z4 hypomethylation and a similar clinical phenotype to FSHD1 [6]. Heterozygous mutations in *DNMT3B* (DNA [cytosine-5-]-methyltransferase 3β) also cause hypomethylation, but only those who have relatively short 9–13 D4Z4 repeats on a permissive A allele exhibit the FSHD phenotype [7]. More recent investigation demonstrated that a homozygous mutation in *LRIF1* (ligand-dependent nuclear receptor interacting factor 1, also called *HBiX1*), a known SMCHD1 protein interactor, can cause hypomethylation of the D4Z4 repeats and produce an FSHD2 phenotype [8]. DUX4 is a transcription factor normally silenced after early development that can trigger a wide range of downstream pathologic consequences in skeletal muscle upon its inappropriate expression.

Facioscapulohumeral muscular dystrophy (FSHD) is among the most common muscular dystrophies, affecting between 1 in 8000 to 1 in 20,000 individuals in different populations [1]. FSHD preferentially involves muscles of the face, shoulder girdle, and those overlying the humerus (biceps and triceps muscles) (Figure 1A). The disease onset and severity vary widely, and affected patients can be categorized into three major groups: (i) early onset (age < 10), which can include the most severe involvement or progression, (ii) typical young-adult onset with variable progression, and (iii) later adult onset. There is also a group of unaffected, asymptomatic carriers of the disease alleles. Although the prevalence is higher in male patients [2], asymptomatic carriers are more often female [3]. More recent investigation has also categorized FSHD patients based on a comprehensive clinical evaluation form (CCEF) into four groups [4]: (i) patients with typical facial and scapular girdle muscle weakness (category A, subcategories A1–A3), (ii) patients with muscle weakness limited to scapular girdle or facial muscles (category B subcategories B1, B2), (iii) asymptomatic/healthy subjects (category C, subcategories C1, C2), and (iv) patients with myopathic phenotype presenting clinical features not consistent with typical FSHD phenotype (D, subcategories D1, D2) [4]. A five-year follow-up study also showed that clinical categories are associated with diverse disease trajectories and these CCEF categories has strong prognostic effect in FSHD1 patients [5].

More recent investigation has also revealed that affected females are more likely overall to progress to wheelchair use and at a faster rate compared to males, independent of genetics [9]. About 95% of patients are classified as autosomal dominant FSHD type 1 (FSHD1), while 5% belong to FSHD type 2 (FSHD2) and have digenic inheritance resulting from an additional genetic defect that contributes to aberrant double homeobox 4 (*DUX4*) gene derepression [10].

Both FSHD1 and FSHD2 result from epigenetic derepression in skeletal muscles of the double homeobox 4 (*DUX4*) gene located at chromosome 4q35 [11,12] (Figure 1B). In affected FSHD1 patients, contraction of the locus to 1–10 repeat units is associated with local DNA hypomethylation and in the presence of a permissive 4qA allele allows *DUX4* to produce a stable polyadenylated transcript. It is noteworthy that 4qA D4Z4 alleles with 4–10 repeats have been detected in 3% of the healthy population [13,14]. *DUX4* encodes a transcription factor that is normally expressed in the germ line, preimplantation embryo, and mesenchymal stromal cells [15]. DUX4 misexpression in mature skeletal muscles activates a large ensemble of target genes that normally function in embryogenesis and germline development, causing muscle cell death [16]. Although the mechanisms leading to *DUX4*-triggered muscle pathology in only a small fraction of muscle cells at one time are incompletely understood, a variety of pathways may contribute to the FSHD phenotype. These downstream mechanisms include either p53-dependent or -independent cell death [17,18], oxidative stress [19], misregulation of the myogenic program and defects in differentiation and fusion [20,21,22], altered cell migration [23], disruption of RNA metabolism including RNA splicing, surveillance and transport pathways [24], altered proteomes and ubiquitination [21,25], aggregation of nuclear proteins (e.g., transactive response DNA-binding protein 43 kDa and fused in sarcoma) [25], formation of double-stranded RNA granules [26], or accumulation of hyaluronic acid causing mitochondrial mislocalization/dysfunction [15].

Pre-clinical research studies have identified an increasing number of candidate therapeutics targeting aberrant *DUX4* expression or activity that soon will be ready for rigorous clinical evaluation. However, several challenges to the implementation of informative FSHD clinical trials remain, including heterogeneity of muscle involvement, rarity of detectable *DUX4* expression, a shortage of robust FSHD biomarkers, and relatively slow disease progression in adult patients. Suitable outcome measures for FSHD clinical trials are critically needed to establish trial design, sample size, and duration within reasonable cost. Many FSHD clinical trials to date have reported measures of muscle function using relatively small sample sizes or short trial durations. As the field prepares for new trials in FSHD, the use of reliable and sensitive longitudinal outcome assessments in trials will be important to maximize statistical power. Herein we discuss the strengths and limitations of current outcome measures used in FSHD trials and natural history studies, and we consider some of the challenges that investigators must overcome.

## 2. Outcome Measures in FSHD Clinical Trials

In general, outcome assessments in FSHD natural history studies or clinical trials are divided into 3 major categories (Table 1). The first category is “clinical outcomes”, which mainly arises from the patients’ assessment in an outpatient clinical setting where the functional status and disease progression can be monitored objectively. Examples of such clinical measures include assessment of muscle strength, timed walk test, reachable workspace (RWS), and FSHD composite outcome measure (FSHD-COM). The second category is “patient-reported outcomes”, which employ patient-reported measurements and questionnaires such as the FSHD Health Index (FSHD-HI) and the FSHD Rasch-built overall disability scale (FSHD-RODS). The third category is “biomarkers”, which are quantitative measures to assess the presence of disease markers or progression, monitor treatment responses, or assess for safety. These can include imaging biomarkers (e.g., ultrasound and magnetic resonance imaging (MRI)), physiological biomarkers such as electrical impedance myography (EIM), tissue biomarkers from muscle biopsies, or biofluid (e.g., blood or urine) biomarkers. In this section, we discuss the potential advantages and challenges for each category of outcome measure in clinical trials and observational studies of FSHD patients.

### 2.1. Clinical Outcomes

#### 2.1.1. Muscle Strength

Manual muscle testing (MMT) is an essential element in physical examination of neuromuscular patients. This can be obtained using the standard Medical Research Council (MRC) scale (first published in 1943 [29]), which scores each muscle strength on a scale of 0 (no muscle movement at all) to 5 (normal muscle contraction against full resistance) [30]. Despite its use in various neuromuscular clinical trials, some limitations of this testing include poor sensitivity [31] and possible inter-rater variability. Besides the MRC scale, muscle strength can be measured by maximal voluntary isometric contraction testing (MVICT) using multiple devices such as fixed or hand-held dynamometry (HHD). These two dynamometry approaches were also shown to provide equivalent results in neuromuscular patients [32]. However, four factors may affect the appropriate collection or interpretation of these data:

- Patient cooperation or effort;
- Ceiling effect: Overall, it is important that the evaluator can overpower the patient’s strength to obtain a valid assessment in HHD [33], although this might be challenging particularly for large muscles such as iliopsoas muscle. Therefore, its sensitivity may be limited when strong muscles are assessed [34].
- Floor effect: If the patient has significant muscle weakness that cannot move against gravity, this may limit dynamometry assessment [35];
- Investigator experience: This important factor is needed to ensure intra- and inter-rater reliability [36];

Both MMT (based on MRC scale) and MVICT yielded highly reliable and valid measures of FSHD state in initial studies (1994) [37,38]. More recently, the two tests were assessed every 6 months for 3 years in a natural history study on 81 FSHD patients [39]. The most affected muscles were proximal arm muscles and foot dorsiflexors, with more homogeneous scores in the shoulder girdle muscles compared to other muscles [39]. A strong linear relationship between the MVICT and MMT scores was present [39]. Moreover, weaker muscle strength scores were overall present in patients with: (i) disease onset age of <20 years and (ii) disease duration of ≥20 years. The mean changes in MVICT and MRC scales were −0.29 and −0.07 at one year, respectively, and followed a similar trend for subsequent follow-up visits [39]. There was no association between these changes and either gender, age, symptoms onset date or duration [39]. A sample size of 160 patients in each arm was calculated to provide 80% power to identify a mean difference of 0.30 points for the MVICT score, or a difference in mean change of 0.07 points for the MMT score [39]. Muscle strength assessment using either MMT or MVICT has been used in several FSHD trials (Table 2) [27,40,41,42,43,44,45]. In a study by Statlend et al. (2013) [46], data from three randomized clinical trials (with albuterol and the myostatin inhibitor drug MYO-029) [47,48,49] and one natural history study [39] totaling 277 patients were analyzed. An increase in strength at 6 months was shown in both MVICT and MMT related to participation in these clinical trials, regardless of being in either the placebo or the active therapy groups, compared with the natural history group [46]. For the MVICT, this effect on strength continued for ~12 months, while for MMT it decreased after 12 months [46]. These results highlight potential placebo effects of participation in FSHD clinical trials.

A Class II study [50] and Class III study [51] have shown that respiratory muscle involvement and abnormal pulmonary function test (PFT) results can develop in FSHD patients, with estimated prevalence between 1.25% and 13% [52]. In a study on 81 FSHD1 patients, none of the ambulatory patients had abnormal PFT, but more than 30% of non-ambulatory patients, especially those with (kypho)-scoliosis, had abnormally restricted PFT (as measured by forced vital capacity [FVC], forced expiratory volume in 1 s, and static maximal inspiratory and expiratory mouth pressures) [53]. Overall, abnormal respiratory volumes have correlated with clinical severity and disability [54,55], higher body mass index [54], and a smaller number of D4Z4 allele repeats [54,55], and expiratory muscles were affected more than inspiratory muscles [56,57]. Although it is recommended that baseline PFT is performed in all FSHD patients, annual PFT is more reserved for those with kyphoscoliosis, significant proximal muscle weakness, wheelchair dependency, or comorbidities affecting ventilation [10,52]. Respiratory insufficiency necessitating nocturnal ventilatory support at home is rare (e.g., ~1% of the Dutch FSHD population) [50,58]. Although the above-mentioned cross-sectional studies have demonstrated variable degrees of respiratory dysfunction in FSHD patients, longitudinal data to assess the progression rate of respiratory dysfunction and PFT changes are lacking. This is an important consideration because longitudinal PFT monitoring is currently used in many clinical trials on neuromuscular patients such as those with amyotrophic lateral sclerosis. Only a few FSHD trials have used FVC as an outcome measure (Table 2) [41,45].

#### 2.1.2. Leg Function: Timed Walk Test and Other Related Measures

The timed walk test is a submaximal exercise test evaluating aerobic capacity, strength, and endurance. The distance covered over a specific period (usually 2, 6, or 10 min) is reported as an outcome to compare changes in performance capacity. The most common measurement is the 6-min timed walk (6-MWT) test, which was initially introduced by the American Thoracic Society (2002) for use in cardiopulmonary trials [59]. The 6-MWT test was gradually applied, even as a primary outcome measure, in trials involving neuromuscular subjects such as Duchenne muscular dystrophy [60]. In a two-center, prospective, cross-sectional study on 86 ambulatory FSHD patients, the mean distance walked during the 6-MWT was markedly impaired compared to healthy control individuals (404.3 versus 571 m, respectively) [61,62] and was not associated with gender and age differences [61]. The minimal detectable change or MDC95 (i.e., the extent of change providing 95% certainty that the change extends beyond measurement error) was 34.3 m [61]. There was also a very strong test-retest reliability for baseline versus <3 weeks assessments (intraclass correlation coefficient (ICC), 0.99 (lower confidence limit of 0.98)) as well as a moderate to strong association with other FSHD disease severity measures including FSHD clinical score, the 30 foot Go, the 10 m/walk run tests, timed up and go (TUG), and lower extremities MMT [61]. However, the test may have low utility in non-ambulatory FSHD patients.

**Table 2:** Outcome measures used in FSHD clinical trials.

Ref	Agent	Outcome Measures	Results of Outcome Measures
Kissel et al. [47]	Albuterol (8 or 16 mg twice daily, 52 weeks)	Primary: 52-week change in MVICT	No significant difference
		Secondary: 52-week change in MMT, grip strength (HHD), functional testing, & muscle mass assessed by DEXA	No significant changes in MMT & functional testing; significant changes in grip strength (HHD) in both doses; significant differences in muscle mass but only in high dose
van der Kooi et al. [48]	26-week strength training of elbow flexors & ankle dorsiflexors followed by 26-week albuterol (8 mg twice daily)	Primary: 52-week change in MVICT	Significant changes only in elbow flexors, but not in ankle dorsiflexors.
		Secondary: 52-week change in handgrip assessed by computer interfaced Jamar grip dynamometer as well as total body skeletal muscle volume estimated by stereologic CT method	Significant improvement
		Other: 52-week change in timed motor performance tasks included standing from lying supine, standing from sitting, walking 30 feet (9.14 m), & climbing three standard stairs	No significant changes
Payan et al. [63]	Salbutamol	Primary: 3- & 6-month change in MVICT	No significant changes
		Secondary: 3- & 6-month change in MMT, QMT, timed motor tests, & scores of the 8 dimensions of the SF36 quality-of-life scale	
Wagner et al. [49]	MYO-029	Primary: Safety & tolerability	Safe & well tolerated
		Secondary: 6-month change in MMT, QMT, timed function tests (time to traverse 9 m, climb 4 stairs, & stand from a seated position), SF36 quality-of-life scale, muscle mass assessed by DEXA & MRI, muscle histology	No significant difference
Walter et al. [41]	Creatine	Primary: 8-week change in MRC scale & NSS	Mild improvement in muscular dystrophy patients in general
		Secondary: Patient’s own assessment of improvement & 8-week change in FVC	Self-reported improvement in 60% of patients with muscular dystrophy in general; no significant change in lung function
Tawil et al. [42]	Prednisone	12-week change in MMT, MVICT, muscle mass assessed by DEXA	No significant change
Sitzia et al. [64]	Flavomega	Primary: Safety & tolerability	Safe & well tolerated
		Secondary: 24-week change in 6-MWT & isokinetic knee extension	Significant change in FSHD/LGMD group (FSHD analysed as a group together with LGMD)
Passerieux et al. [43]	Supplement	Primary: 17-week change in 2-MWT, MVCQD, MVCQND, TlimQD, & TlimQND	Significant change in MVCQ and TlimQ; no significant change in 2-MWT
		Secondary: 17-week change in serum oxidative stress markers (vitamin C, α-tocopherol, vitamin C/vitamin E ratio, vitamin E γ/α ratio, & lipid peroxides)	Significant improvement
van der Kooi et al. [65]	Folic acid & methionine	Primary: 12-week change in methylation level in blood lymphocytes	No increase in methylation
ReDUX trial (NCT04003974) [27]	Losmapimod	Primary: 48-week change in *DUX4*-driven gene expression in muscle biopsies	No significant change detected; high variability
		Secondary: 48-week change in TUG, FSHD-TUG, muscle dynamometry, RWS, MFM domain 1, PGIC, FSHD-HI, and muscle MRI signal properties	Significant improvement in RWS & PGIC, decreased progression in muscle fat infiltration in active versus placebo group; no significant change in TUG, FSHD-TUG, overall dynamometry, MFM domain 1, & FSHD-HI
Gershman et al. [40]	ATYR1940	Primary: Safety & tolerability	Safe & well tolerated
		Secondary: Changes in INQoL, lower extremity muscle targeted MRI, & MMT	Significant dose-dependent improvement in INQoL; no significant change in MRI finding; no reportable disease progression in MMT of all groups
Statland et al. [66]	ACE-083	Primary: Safety & tolerability	Safe & well tolerated
		Secondary: 3-month change in muscle mass & intramuscular fat fraction in Dixon MRI	Significant increase in total muscle volume & intramuscular fat fraction with ACE-083 treatment
NCT02927080 [44]	ACE-083	Primary: 190-day change in muscle mass & intramuscular fat fraction in Dixon MRI	Significant increase in total muscle volume
		Secondary: 190-day change in TA function (6-MWT, 10 m walk/run & 4-stair climb/ascend), biceps strength (HHD & MVIC), PUL of mid-level elbow dimension, & FSHD-HI	No significant change in TA function, PUL assessment, & FSHD-HI; significant change in biceps strength
STARFISH trial (NCT03123913) [45]	Testosterone and rHGH	Primary: Safety & tolerability	Ongoing study
		Secondary: 24-week change in 6-MWT, MMT, FVC, QMT, & FSHD-HI	

2-MWT, 2-min walk test; 6-MWT, 6-min walk test; DEXA, dual energy x-ray absorptiometry; DMD, Duchenne muscular dystrophy; FSHD-HI, FSHD-health index; FVC, forced vital capacity; HHD, hand-held dynamometer; I.M., intramuscular; INQoL, individualized neuromuscular quality of life questionnaire; LGMD, limb girdle muscular dystrophy; MFM, motor function measurement; MMT, manual muscle testing; MRC, medical research council; MRI, magnetic resonance imaging; MVICT, maximum voluntary isometric contraction testing; MVCQD, maximal voluntary contraction of the dominant quadriceps; MVCQND, maximal voluntary contraction of the nondominant quadriceps; NSS, neuromuscular symptoms score; PGIC, patients’ global impression of change; PUL, performance of the upper limb; QMT, quantitative muscle testing; rHGH, recombinant human growth hormone; RWS, reachable workspace; TA, tibialis anterior; TlimQD, limit time of the dominant quadriceps; TlimQND, limit time of the nondominant quadriceps; TUG, timed up and go.

The TUG test assesses ambulation and balance when the patient stands from a chair, walks three meters at normal pace, turns, walks back at normal pace, and sits down again. Although it was originally developed for elderly patients, it is currently used as a reliable measure for different neurological diseases such as Parkinson’s disease [67]. The 30 foot Go test is a test of maximal performance measuring pace and strength where individuals are requested to traverse 30 feet as fast and safe as they can. The timed 10 m walk/run is also similar to this test, but it is done over 10 m [68]. The time to ascend 4 stairs mainly assesses proximal leg strength [69]. A previous FSHD history study found that the functional motor tests (i.e., timed 30 foot Go, the time to get up from a chair, and time to ascend 4 stairs) were reliable and had good cross-sectional relations to the disease severity, but they were not separately sensitive to the FSHD progression [37,39]. In two recent studies, instrumented TUG (iTUG) with wireless inertial motion sensors was assessed in FSHD patients and the results demonstrated that the iTUG was abnormal in FSHD, reliable, and sensitive to the disease severity and progression [70,71]. Several FSHD trials have recently utilized 2-MWT [43], 6-MWT [44,45,64], TUG [27], timed 10 m walk/run [44,48], and time to climb/ascend 3 or 4 stairs [44,48,49] as one of their outcome measures. None of the completed trials (ranging between 17 to 52 weeks) showed a significant change in any of these measures (Table 2).

#### 2.1.3. FSHD Clinical Score

In 2010, the FSHD Clinical Score or evaluation score [72] was generated to quantify clinical severity by examining characteristically affected muscle groups in FSHD. The FSHD Clinical Score consists of 6 separate sections evaluating the strength and function of the following muscle groups with the total score between 0 (no muscle weakness) and 15 (when all muscles are severely weak): (i) facial muscles (total score 2), (ii) scapular girdle muscles (total score 3), (iii) upper limb muscles (total score 2), (iv) distal leg muscles (total score 2), (v) pelvic girdle muscles (total score 5), and (vi) abdominal muscles (total score 1). The FSHD Clinical Score had no significant variation dependent on the examiner, considering single items and the overall good inter-rater kappa value of 0.7744 (i.e., degree of agreement among physicians’ assessments) [72]. The FSHD clinical score was later used in some cross-sectional studies on FSHD patients [73,74] as well as a recent 5-year observational study on 246 FSHD1 patients [5]. The latter showed that the FSHD Clinical Score varies through time and the increment of the FSHD score can define a disease trajectory with variable steepness across the four clinical categories described by the CCEF [5].

#### 2.1.4. Reachable Workspace (RWS)

The 3D Kinect-based RWS is a test that was developed in 2015 to evaluate shoulder girdle function [74]. In this test, a subject sits in front of the Microsoft Kinect sensor and performs a standard upper limb movement protocol including raising the arm from the resting position to above the head while keeping the elbow extended, performing the same movement in vertical planes at around 0, 45, 90, and 135 degrees as well as horizontal sweeps at the umbilicus level and shoulder [74]. This test is feasible and reliably abnormal in FSHD patients in comparison with healthy subjects, and is also sensitive to the disease severity [74] when compared to the FSHD clinical score [72]. Furthermore, the quantitative MVICT of shoulder abduction and elbow flexion moderately correlates with RWS [75]. Based on a more recent investigation, RWS moderately to strongly correlates with daily life activities at home (especially pick-up clothes, shirt on, shirt off, use spoon, and pull on pants) [76]. Notably, the recent randomized, double-blind, placebo-controlled (phase 2b) trial of Losmapimod (an inhibitor of p38α/β mitogen-activated protein kinases) on 80 FSHD patients found small but significant improvements of up to 1.5% in RWS surface area after 48-week treatment with Losmapimod compared to placebo (Table 2) [27].

#### 2.1.5. FSHD Composite Outcome Measure (FSHD-COM)

The FSHD-COM was developed in 2018 to provide an evaluator-administered comprehensive functional assessment of patient-identified areas of functional burden for upcoming clinical trials [77]. In this 18-item instrument, each item has a rating between 0 (unaffected/normal performance) and 4 (severely impaired/unable to complete) with the total scale of 72 points. Five major body regions functions are assessed:

- Leg function (total 24 points): Six continuous measures (sit to stand, 6-MWT, self-selected gait speed, 30 foot Go, and ascend/descend stairs) are evaluated and converted to a 0–4 ordinal scale using divisions. The combination of these measures helps evaluators assess different domains of leg function, including power, endurance, balance, and climbing;
- Shoulder and arm function (total 28 points): This consists of 7 measures (bilateral shoulder abduction, shoulder forward flexion, elbow flexion, and don/doff coat) assessing proximal to mid arm tasks, and the power to coordinate upper limb function across various muscle groups;
- Trunk function (total 12 points): In this part, practical tasks reflecting changes in core truncal muscles are examined, which include picking a penny up off the floor, sitting up from lying supine, and the timed supine to sitting at edge of bed;
- Hand function (total 4 points): In this part, hand grip strength is measured bilaterally;
- Balance/mobility (total 4 points): TUG is timed and then scored from 0 to 4.

The FSHD-COM has demonstrated a very strong test-retest reliability (ICC, 0.96) [77] and moderate to strong correlation with the muscle strength, disease severity, and duration [77]. A recent study on eighteen children with FSHD also found that the FSHD-COM and its modified pediatric version showed excellent intra-rater reliability (ICC_1,2_ > 0.99, lower 95% CI > 0.98) with a Minimal Detectable Change (MDC95%) of ≤14.5% [28]. The FSHD-COM had very strong correlations with other related outcome measures including MFM-32, FSHD Severity Scales, Performance of the Upper Limb 2.0, Pediatric Quality of Life™ Neuromuscular Module and pediatric FSHD-HI questionnaire [28]. Previous clinical trials have not used the FSHD-COM; however, an ongoing 18-month multicenter natural history study on 160 FSHD patients (ReSolve) is currently using this as a primary outcome measure [78].

#### 2.1.6. Ricci Clinical Severity Score (CSS or Ricci Score)

In 1999, Ricci et al. developed the Clinical Severity Score (CSS, also called Ricci score) for FSHD patients to describe the clinical severity of the disease [79]. It is a 10-grade severity score that considers the extent of weakness in various body regions, in which 0 implies no muscle weakness and 10 implies wheelchair dependency. The score takes into account the descending spread of symptoms from face and shoulders to pelvic and leg muscles, which is typical for FSHD patients [79]. Thus, patients in whom pelvic and proximal lower extremities muscles are affected have higher scores. Given the variability of disease onset age in FSHD, the CSS was further adjusted by age with the following formula [80]:

Age-corrected CSS = ((CSS × 2)/age at examination) × 1000

However, the assumption of a fixed sequence of muscle involvement makes this score less suitable for FSHD patients with an atypical disease course; thus, this score has been substituted to some extent with other scales (e.g., FSHD Clinical Score) in clinical studies on FSHD patients [81].

#### 2.1.7. Motor Function Measure (MFM)

The MFM is another reliable and sensitive evaluator-reported scale that can measure motor deficit severity and progression in various neuromuscular diseases. It comprises 32 items in 3 functional domains of (i) standing position and transfers, (ii) axial and proximal motor function, and (iii) distal motor function [82,83,84,85]. The MFM domain 1 was recently used in the Losmapimod trial with no significant changes seen in active versus placebo groups [27]. More studies using larger number of patients and longer duration are needed to assess its benefit in FSHD trials or natural history studies. 

#### 2.1.8. Remote Monitoring Functional or Motor Outcomes

Recent advances in sensor technology and digital health tools have undoubtedly helped health care providers and investigators to assess disease progression and recovery in a clinical setting as well as outcome measures in trials in a variety of neurological conditions including neuromuscular diseases [86,87]. This has also been helpful in more efficient assessment or management of these patients remotely during televisits [88]. In 2016, Gidaro et al. [89] introduced ActiMyo (a home-based activity recording device with two sensors) to assess ambulant and non-ambulant patients via recording different types of movements of the upper and/or lower extremities during daily life activity. Their pre-liminary data on 9 FSHD patients who wore ActiMyo at the dominant wrist and the ipsilateral ankle during the 6-MWT revealed that in all patients tested, the difference between the distance measured by the clinician and the distance calculated by the ActiMyo was less than 3% [89]. Subsequent study on patients with FSHD and limb-girdle muscular dystrophy type 2B (LGMD2B), who were part of the ATYR1940 clinical trial, showed the feasibility of monitoring gait activity at home in 10 ambulant patients using ActiMyo and found its utility in detecting statistically significant mild decreases in median step speed over a 4 month period [90,91], suggesting that home-recorded stride speed constitutes a precise and sensitive outcome in ambulant patients with FSHD and LGMDR2B. A more recent study [92] also utilized two digital technologies (CHDR MORE and Withings Health applications) for 6 weeks to assess social and physical activity and to evaluate their correlation to FSHD Clinical Scores (i.e., Lamperti scores) and the TUG in 38 FSHD patients compared to 20 control subjects. The study demonstrated correlation of a composite of sleep quality, app use, location, and physical activity with Lamperti scores (R2: 0.54; mean square error (MSE): 4.89) and a composite of sleep quality, app use, location, physical activity and texting behavior with TUG (R2: 0.85; MSE: 1.0) [92]. Overall, these results suggest the utility of digital biotype to quantify disease severity in FSHD patients and potentially to monitor disease progression.

### 2.2. Patient Reported Outcomes

#### 2.2.1. FSHD Rasch-Built Overall Disability Scale (FSHD-RODS)

Considering the World Health Organization international classification of disease-related functional consequences, a very recent multi-center study on 762 FSHD patients in 5 countries (The Netherlands, UK, USA, France, and Australia) introduced the FSHD-RODS, which is a 32-item, patient-reported interval assessment of various aspects of daily activity and participation restrictions in FSHD patients [93]. This scale has shown a good discriminative validity when correlated with the MFM with a Spearman’s correlation coefficient of 0.86 [93]. Moreover, a robust internal reliability for the FSHD-RODS (person separation index of 0.97) as well as excellent test-retest reliability scores were observed [93]. In contrast to previous FSHD outcome measures, which are nearly all ordinal-based measures, a major benefit of the FSHD-RODS is that it provides an interval scale, allowing parametric testing and comparison changes throughout the scale [93,94]. Another potential benefit of FSHD-RODS is the ease of longitudinal measurement and reporting by patients. Cross-cultural validation as well as further longitudinal studies are needed for the application of FSHD-RODS in clinical practice or future trials.

#### 2.2.2. FSHD Health Index (FSHD-HI)

The 116-item FSHD-HI was originally designed in 2012 to identify the symptoms of highest importance and burden based on extensive clinic interviews with 20 adult FSHD patients [95]. Four major quality of life domains (physical, mental, and social health, and FSHD specific issues) consisting of a total of 14 subdomains are reported upon by FSHD patients [95]. A subsequent study on 22 individuals showed an excellent test-retest reliability (ICC, 0.945) [95,96]. FSHD-HI responsiveness is being assessed in the longitudinal ReSolve study [97], and it may be sensitive to measure small disease burden changes over 6 to 12 months of observation [96]. Additionally, FSHD-HI has been used in previous FSHD trials (e.g., Losmapimod [27] and ACE-083 [44]) in which neither intervention produced significant changes in FSHD-HI [27,44]. FSHD-HI is also currently being used as a secondary outcome in an ongoing trial on testosterone and recombinant human growth hormone (rHGH) in FSHD [45].

#### 2.2.3. Activity Limitations (ACTIVLIM) Questionnaire

ACTIVLIM is a Rasch-built, 22-item, patient-reported measure that was developed in Belgium to assess daily living activities’ limitations in neuromuscular patients with upper and/or lower limb impairments [98]. The perceived difficulty in performing each item is scored between 0 to 1 (impossible, difficult, and easy, respectively). In a multi-center cohort of 2986 neuromuscular (including FSHD) patients over 2 years, ACTIVLIM confirmed excellent fit to a unidimensional scale, exhibited a good reliability (R = 0.95) and capability to quantify small but significant changes in activity for various diagnostic groups [99]. ATIVLIM has been recently utilized as an outcome measure in some neuromuscular clinical trials including adult patients with spinal muscular atrophy types 3 and 4 [100] and late-onset Pompe disease [101]. Thus, it has potential to be utilized in future FSHD clinical trials or natural history studies.

### 2.3. Biomarkers

#### 2.3.1. Imaging Biomarkers: Muscle MRI and Ultrasound

Muscle MRI has been used as a diagnostic tool especially for inherited or acquired myopathies that show characteristic patterns of muscle involvement (i.e., preferentially involved and preferentially spared muscles) that can be detected using established MRI pulse protocols. As MRI is a non-irradiating, non-invasive tool with high spatial resolution that is not generally biased by patient or investigator efforts as well as clinical symptom variability, it may be considered an attractive outcome measure in neuromuscular trials. In general, the following features in muscle MRI can be assessed:

- Fatty tissue replacement or infiltration of muscle: This reflects either remote damage or muscle fiber loss, measured by the increased signal intensities in the T1-weighted images. Compared to fatty tissue infiltration, endomysial fibrosis might serve as a complementary marker for loss of muscle function, but current MRI protocols do not show sufficient sensitivity to quantify fibrosis [102].
- Ongoing muscle damage, including edema or inflammation: the short-tau inversion recovery (STIR) or T2-weighted images can detect increased water content or edema, which may reflect ongoing muscle damage or inflammation [102].
- Fasciitis and mass lesion: These abnormalities are usually detected in post-contrast sequences, which are not routinely used for inherited myopathies due to its limited diagnostic utility.
- Muscle volume (atrophy or hypertrophy): This can be assessed for individual muscle groups by the T1-weighted images and quantitative MRI analyses.

Whole-body MRI has also provided valuable information on the more affected muscles in FSHD patients [103,104,105,106]. While great variability occurs in FSHD, some muscles that may be difficult to isolate clinically on strength testing (e.g., the hamstring semimembranosus muscle) can frequently show abnormal MRI signal characteristics in FSHD. Along with the rectus abdominis muscle, the frequency of affected muscles in these studies are as follows [10,102,104,107,108,109]:

- The semimembranosus muscle: >70%
- The paraspinal and serratus anterior muscles: 60–70%
- The trapezius, adductors, latissimus dorsi, and gluteus minimus muscles: 50–60%.

Iliacus, iliopsoas, peroneus, and tibialis posterior muscles are the least affected muscles in FSHD [10,102,103,104,107,108]. Muscle abnormalities can also be detected using MRI in individuals with asymptomatic FSHD [103,110].

Although an initial 6.9- to 13.8-month, longitudinal prospective study on 15 FSHD patients showed no significant progression of fatty tissue infiltration in most muscles (likely due to the slowly progressive nature of FSHD) [111], a recent study on 36 patients revealed that muscles with intermediate baseline fat fraction had more likelihood of progression with follow up MRI at one year [108]. Although there was no significant change in functional outcomes (i.e., 6-MWT or 30 foot Go), overall MRI disease burden correlated with these measures [108]. Additionally, the fat fraction in the TA muscle showed a sigmoid association with foot dorsiflexion strength measured by quantitative HHD, with steepest decline when the muscle had >20% fatty replacement [108]. Other longitudinal studies have also indicated that quantitative MRI is more responsive to detect changes than most traditional muscle tests [112,113,114]. STIR hyperintensity (i.e., edema suggestive of active inflammation) is observed to develop before end-stage irreversible T1-weighted hyperintensity (i.e., fatty tissue replacement) [114,115]. Such inflammatory regions may represent more active disease progression and their extent could be an important focus in clinical trials [116]. MRI quantitative volumetric assessments and fatty infiltration have been outcome measures in previous trials of MYO-029 [49], ATYR1940 [40], and ACE-083 [44,66]. The recent 48-week Losmapimod trial in 80 FSHD subjects also found a significant improvement in fat infiltration of some affected muscles with Losmapimod compared to placebo [27]. Notably, this study also utilized MRI STIR hyperintensity as a guide for muscle biopsy, as previous investigations have found that active inflammatory cell infiltration may be present in these areas [110,117,118].

Although muscle ultrasound is not a routine diagnostic tool for myopathies or muscular dystrophies, it may provide a useful research biomarker, since ultrasound is a rapid, painless, non-invasive, non-irradiating, and cost-effective procedure. Limitations of muscle ultrasound include lower image resolution compared to MRI, sensitivity to assess superficial rather than deep muscles, and technician/physician technique variation. Nevertheless, ultrasound can provide valuable information regarding muscle echogenicity, atrophy, and architectural changes due to fibrosis or fatty degeneration [119]. Recent studies have also utilized dynamic muscle ultrasound to assess normal/abnormal muscle contraction or deformation patterns in muscular dystrophies and even fasciculations in motor neuron diseases [119]. Studies on patients with some muscular dystrophies have shown good correlations between muscle ultrasound findings and disease progression or functional performance over time [120,121].

Limited studies have assessed muscle ultrasound in FSHD. In the first published study, quantitative ultrasound and MRI were assessed in two lower extremity muscles (vastus lateralis and rectus femoris) of five patients [122]. The echo intensity z-score in quantitative muscle ultrasound strongly correlated with muscle fraction and T1 signal intensity in quantitative MRI [122], with a wider dynamic range for quantitative muscle ultrasound making it a more helpful tool in the follow-up of advanced disease stages. In a subsequent proof-of-principle study, dynamic ultrasound was applied to assess the TA muscle deformation in 4 FSHD patients compared to healthy controls [123]. A markedly reduced motion of the central tendon aponeurosis of the TA muscle was noted in the severely affected muscles compared to less affected or healthy ones [123]. The measured force also correlated well with the muscle motion (linear coefficient of determination or R^2^ value of 0.91) with an additional finding of the highest quantitative ultrasound echogenicity z-scores (2.7–4.78) for severely affected TA muscles [123]. In another study on 27 FSHD patients, fatty infiltration, fibrosis, and edema were assessed by both quantitative ultrasound and quantitative MRI in 10 leg muscles [124]. There was a strong correlation between the MRI fat fraction and ultrasound echogenicity z-score (CC 0.865); both correlated robustly with clinical severity (ultrasound CC 0.767 and MRI CC 0.828) [124]. However, MRI was more sensitive than ultrasound in identifying muscle edema or late stages of fatty infiltration, emphasizing that the two techniques are complementary [124]. Considering that TA is frequently affected and peroneus longus is among the least affected muscles in MRI and clinical studies of FSHD [103,107], a recent study performed qualitative and quantitative assessments of muscle echogenicity [125] in both TA and peroneus longus muscle of 8 FSHD1 patients. The study found a clear pattern of preferential TA involvement and peroneus longus sparing in all patients, with additional finding of a strong correlation between TA hyper-echogenicity and muscle weakness (MRC scale) in patients with only mild to moderate (and not severe) muscle weakness [125].

So far, only one longitudinal study has assessed both quantitative and qualitative muscle ultrasound changes in FSHD [126]. In this one-year observational study on 22 FSHD patients, 5 muscles (TA, biceps, rectus femoris, rectus abdominis, and trapezius) were assessed at baseline and after 12 months with quantitative and qualitative muscle ultrasound as well as clinically with MRC scale, adjusted CSS, and the FSHD Clinical Score [126]. The results demonstrated that although the MRC scales did not change, both the qualitative ultrasound sum score and quantitative ultrasound sum z scores significantly increased over one year. Notably, the study provided Class I evidence that muscle echogenicity correlated with the baseline FSHD Clinical Score [126], supporting the use of muscle ultrasound as a potential responsive marker in the future clinical trials.

#### 2.3.2. Physiologic Biomarkers: Electrical Impedance Myography (EIM)

EIM is a non-invasive, painless technique that applies multi-frequency, low-intensity alternating electrical currents through the surface electrodes on skin in order to assess the resistance to current flow through a specific muscle [127]. Since muscle has different impedance to current flow compared to other tissues (especially fat), EIM may estimate changes in muscle composition. EIM can provide good reliability, responsiveness to disease progression, or association with disease severity in a variety of neuromuscular diseases [128,129,130]. EIM was evaluated in 35 adult FSHD patients and exhibited a good to excellent reliability for deltoid, biceps, triceps, vastus lateralis, and tibialis anterior (TA) muscles (ICCs range: 0.72–0.99), acceptable reliability for the abdominal muscles and thoracic paraspinal muscles (ICCs range: 0.71–0.98), and less reliability for facial muscles [73]. Notably, the 50 kHz reactance moderately to strongly correlated with other FSHD disease measures including muscle strength, time to ascend 4 stairs, and the 6-MWT [73]. EIM measures (resistance, reactance, and phase at 50, 100, and 211 kHz) also correlated with MRI T1-based muscle severity score and MRI quantitative intramuscular Dixon fat fraction [131]. However, another study on 32 FSHD patients found EIM not sensitive enough to identify significant disease progression over one year [132]. The 18-month prospective ReSolve study is currently assessing the EIM responsiveness to FSHD progression [97].

#### 2.3.3. Muscle Biopsy Biomarkers: Histopathology and DUX4 and Target Gene Expression

With advances in genetic testing, muscle biopsy is now rarely used for making the diagnosis of FSHD. Abnormal findings on muscle biopsy can be quite variable and localized. Nonspecific myopathic features including fiber size variation, internalized or rounded nuclei, rare necrotic or atrophied fibers are present in affected muscles [133]. Additionally, endomysial/perivascular inflammation with CD4 or CD8 T cells are observed in ~30% of cases [117,133], which is compatible with hyperintensity signals in STIR MRI [110,134]. On the other hand, fibrosis and fatty infiltration are seen in more severely affected muscles. Although the above inflammatory markers may be indicative of a step prior to the irreversible fatty replacement and serve as a potential target in clinical trials, they are sporadic and unpredictable, fluctuate, and do not necessarily correlate with the disease severity [109,114]. Moreover, inflammation in some affected muscles recovers without subsequent fatty replacement, and even fatty replacement can happen in the non-inflamed muscles [135]. Therefore, it is still a challenge to consider changes in inflammatory markers in FSHD muscle biopsy as a marker for response to therapies.

Given recent advances in therapeutic gene delivery approaches, the expression dynamics of the *DUX4* gene or its protein product in skeletal muscle could be an important indicator in clinical trials. However, an essential challenge is the detection of *DUX4* transcripts or DUX4 protein in FSHD muscle biopsies [136,137]. Although some studies using highly sensitive nested real-time quantitative polymerase chain reaction (RT-qPCR) have detected *DUX4* expression in FSHD muscle biopsies [138], others using a variety of techniques (e.g., RNA, single cell RNA) have failed to detected it reliably [139,140,141]. Additionally, the cellular or tissue distribution of DUX4 protein and its timeline during differentiation is still elusive. While Western blot analysis of FSHD muscle biopsies detected DUX4 protein, the expression was not found in more severely affected muscles [142], suggesting the possibility that *DUX4* expression in an affected muscle region might only occur early and transiently to trigger subsequent pathology [142,143]. In a recent cross-sectional study on 36 FSHD patients that examined the correlation between lower extremity MRI characteristics, muscle pathology and expression of *DUX4* target genes, it was found that STIR positive muscle MRI measures exhibited a substantial predictive value for identifying muscles with *DUX4* expression and active disease [116]. RNA sequencing of the previously identified four-candidate biomarkers (*TRIM43* (Tripartite Motif-Containing Protein 43), *LEUTX* (leucine 20 homeobox), *PRAMEF2* (PRAME Family Member 2), *KHDC1L* (KH Domain Containing 1 Like)) [141] were performed in the MRI STIR positive muscle biopsy samples compared with the STIR negative control samples, and it was found that the expression of all these markers were elevated in 71% of FSHD samples [116]. The investigators found that using an elevated STIR rating to select muscles with increased *DUX4* target expression would yield positive results in ~90% of the samples [116].

The recent Losmapimod trial attempted to assess the 48-week change in muscle *DUX4*-driven gene activity but found too much variability between longitudinal samples to identify possible changes in the expression of *DUX4* target genes [27]. Recent studies have discovered several signaling pathways that are disrupted in FSHD muscle biopsies, including tumor necrosis factor (TNF)-α signaling, hypoxia-inducible factor 1α over-activation, Wnt/β-catenin signaling, and mitochondrial function via oxoglutarate dehydrogenase L [135,144].

#### 2.3.4. Biofluid Biomarkers: Immune and miRNA Biomarkers

Given the presence of inflammation and infiltration of immune cells, including CD4 or CD8 T cells [110,134] especially in MRI STIR positive muscles in FSHD patients, Frisullo et al. (2011) [117] evaluated the presence of circulating activated immune cells and cytokines levels in FSHD patients with or without STIR positive muscles and from controls. They found higher CD8^+^pSTAT1^+^, CD8^+^T-bet^+^ T cells and CD14^+^pSTAT1^+^, CD14^+^T-bet^+^ cells percentages and IL-12p40, interferon (IFN)-γ and TNF-α levels in FSHD patients with hyperintensity features in one or more muscles compared to patients without muscles STIR hyperintensity and controls [117]. A subsequent prospective, cross-sectional study on 22 FSHD patients compared to 23 control subjects using a commercial multiplex, microsphere-based immune-fluorescent assay of 243 markers also identified 7 serum biomarker candidates including creatine kinase MB fraction, tissue-type plasminogen activator, myoglobin, epidermal growth factor, chemokine (C-C motif) ligand 2, CD40 ligand, and vitronectin [145]. Petek et al. (2016) [146] utilized the SomaLogic proteomics platform of 1129 serum proteins to identify proteins with levels that correlate with FSHD severity in a cross-sectional study of two independent FSHD cohorts (the Rochester cohort and the Seattle cohort). They found 35 proteins in the Seattle cohort and 21 proteins in the Rochester cohort with at least a 1.5 fold difference between FSHD-subjects and controls [146]. Notably, levels of creatine kinase MM and MB isoforms, carbonic anhydrase III, and troponin I type 2 reliably predicted the disease state and correlated with disease severity in both cohorts [146]. Other studies have also suggested serum interleukin 6 [147] or STIR positive muscles’ chemokine (C-X-C motif) ligand 13 (CXCL13) expression [148] as potential FSHD biomarkers. Clearly more studies with larger sample sizes, longitudinal evaluation, and increased diversity of disease severity are needed to verify the value of these biomarkers as outcome measures in FSHD studies. Notably, a recent study profiling serum antibody against muscle antigens in 138 FSHD patients did not find any disease-specific autoantibodies compared to control subjects [149]. *DUX4*-related microRNA signatures in both FSHD skeletal muscles and serum have also been recently investigated [150]. An increased expression of *miR-31-5p* and *miR-206* in muscles of *DUX4*-induced FSHD-like mouse model as well as increased *miR-206* expression in serum of FSHD patients was observed [150].

## 3. Conclusions and Perspectives

Over the last two decades, remarkable progress in our understanding of FSHD pathogenesis has opened a new avenue for translational research studies that effectively target *DUX4* and the underlying pathways contributing to muscular dystrophy in this debilitating disease. However, the design and implementation of FSHD treatment trials must account for patient heterogeneity, a need for more sensitive biomarkers, and the relatively slow disease progression in adult patients. Reliable and practical outcome measures especially in phase II trials should be a focus of investigation in the upcoming years. In addition, the specific clinical phenotype in FSHD diagnosis should be considered. Many studies have reported a large phenotypic variability among individuals carrying a contracted D4Z4 allele [151,152,153,154], implying that a positive molecular test is insufficient for categorizing FSHD [52]. Thorough clinical characterization is at the basis of trial readiness and essential for trial recruitment. The longitudinal study conducted by Vercelli et al. [5] demonstrates that diagnostic decision-making, prognosis, genetic counseling, and patient stratification cannot rely on the molecular testing alone. The family status of the patient (index case or relative), clinical status (classic FSHD, incomplete FSHD, complex FSHD, and non-penetrant cases), and the size of the D4Z4 contracted allele (1–3 versus 4–10 repeats) must be considered to offer appropriate care [5]. Additionally, disease progression varies between index cases and relatives, and assessment of the categories based on the CCEF has a strong prognostic effect in carriers of the FSHD1 molecular signature [5].

As reviewed above, there are three main categories of outcome measures being utilized in FSHD clinical studies, including patient-reported outcomes, evaluator clinical assessments, and biomarkers (e.g., imaging, physiological, muscle, and biofluid biomarkers) (Table 1). Although development of interval scales such as the FSHD-RODS may help to obtain information from the patients’ perspective regarding disease progression, these measures should be complemented by objective measures in clinical trials. As discussed above, conventional methods such as MRC scores may also be subject to biases. Recent new applications of available technology such as the RWS are helping researchers to overcome these biases to some extent. Continued development of objective clinical assessment tools and advanced techniques/devices is essential to reliably evaluate and monitor functional outcomes (e.g., muscle activity, range of motion, and muscle strength) in multiple affected muscle groups and to detect small changes over time. Non-invasive tests (e.g., muscle MRI, ultrasound, and EIM) have yielded invaluable information about the disease characteristics in natural histories; it is hoped that these sensitive tests may find a place in FSHD therapeutic trials if they can be correlated to other functional outcomes. Optimistically, effective therapies applied during the earliest stages of muscle involvement might prevent clinical progression or even improve muscle function, but the challenges of variability in disease location and progression need to be overcome. Given these clinical challenges and the anatomical complexity of FSHD muscle pathology, the identification of circulating molecular biomarkers that can systemically monitor active muscle disease will be an essential tool to be used in combination with whole body MRI and comprehensive quantitative clinical assays of upper body mobility and leg muscle function. Research on FSHD pathophysiology and disease mechanisms is rapidly advancing towards the discovery of validated disease biomarkers and clinical assays to enable clinical trials of new FSHD therapeutics now emerging from pre-clinical research, with promise for treatment of this debilitating disease.

## Figures and Tables

**Table 1:** Overview of outcome measures used in clinical trials on facioscapulohumeral muscular dystrophy (FSHD) and their advantages or limitations.

Category	Examples of Outcome Measures	Advantages	Limitations
Clinical outcomes	Muscle strength (MRC, MVICT, MVCQD, MVCQND)	Non-invasive, rapid, and relatively feasible in clinical setting	Potential biases due to patient cooperation/effort, ceiling effect, floor effect, and investigators’ experience
	Leg function and other related functions (Timed walk test [2, 6-, or 10-MWT], Timed 10 m walk/run test, Timed 300 foot Go, Time to get up from chair, Time to ascend/climb 3 or 4 stairs, Timed up and go [TUG])	Non-invasive, rapid, relatively feasible in clinical setting	May not be informative as a sole reliable outcome for therapy response in trials (i.e., completed FSHD trials do not detect change in these measurements)
	Ricci clinical severity score (CSS or Ricci score)	Feasible and rapid method to assess clinical severity on a 10-point scale	It assumes a fixed sequence of muscle involvement and is less suitable for patients with an atypical disease course
	MFM	Feasible, acceptable, and relatively rapid method of motor functional status assessment	One FSHD trial has used it [27]; more studies are needed to assess its benefits in trials.
	FSHD clinical score	Feasible evaluation of clinical severity with good degree of agreement among physicians’ assessments	Longitudinal studies to assess its correlation to disease progression needed.
	Reachable workspace (RWS)	Feasible and reliable test to assess shoulder girdle function. Recent Losmapimod trial [27] has found its significant change with intervention	Needs equipment in a prepared clinical setting
	FSHD-COM	Reliable test to assess different body regions’ function. Valid and reliable in pediatric FSHD patients [28].	Relatively time-consuming test in a clinical setting; not used in any trials yet; its correlation to disease progression not tested yet in adult patients.
Patient-reported outcomes	FSHD-RODS	Feasible, valid, and comprehensive assessment of various aspects of daily activity and participation restrictions in FSHD patients; it provides interval scale	Limited longitudinal studies to assess its correlation to disease progression; no trial has used it yet.
	FSHD-HI	Comprehensive and reliable assessment of different domains of quality of life and disease burden; used in several trials	Relatively time-consuming test in a clinical setting; limited longitudinal studies to assess its correlation to disease progression
	PGIC	Reliable, subjective assessment of patients feeling about therapy in trials	Not useful as a sole outcome for assessment of response to therapy in trials
Imaging biomarkers	Magnetic resonance imaging (MRI)	A sensitive and objective assessment of muscles involvement and its progression; detects affected muscles prior to development of clinical weakness; useful guide for muscle biopsy; used in trials	Expensive method for disease or therapy response monitoring; not tolerable by all patients; improvement in MRI scores does not necessarily correlate with functional improvement in FSHD trials
	Muscle ultrasound	Rapid, painless, cost-effective procedure to assess affected muscles; It correlates with MRI findings and disease severity	Limited mostly to superficial than deep muscles; needs equipment and experienced staff; longitudinal studies to assess its correlation with disease progression is limited; not tested in trials
Physiologic biomarker	Electrical impedance myography (EIM)	Rapid, painless, cost-effective procedure	Limited data on its sensitivity to disease progression; limited to superficial muscles
Muscle biomarkers	Histopathology, *DUX4* expression or related biological signaling	Potentially good source to assess several target pathways in affected muscles	Invasive and uncomfortable procedure; limited utility due to heterogeneity of muscles involvement or *DUX4* expression
Biofluid biomarkers	Immune and miRNA biomarkers	Potentially good source to assess several target pathways	Limited data

FSHD-COM, FSHD Composite Outcome Measure; FSHD-HI, FSHD Health Index; FSHD-RODS, FSHD Rasch-built Overall Disability Scale; MFM, motor function measurement; miRNA, microRNA; MRC, medical research council; MVICT, maximum voluntary isometric contraction testing; MVCQD, maximal voluntary contraction of the dominant quadriceps; MVCQND, maximal voluntary contraction of the nondominant quadriceps; PGIC, patients’ global impression of change.

## References

[1] Deenen J.C.W., Arnts H., van der Maarel S.M., Padberg G.W., Verschuuren J.J.G.M., Bakker E., Weinreich S.S., Verbeek A.L.M., van Engelen B.G.M. (2014). Population-based incidence and prevalence of facioscapulohumeral dystrophy. Neurology.

[2] Ricci G., Scionti I., Sera F., Govi M., D’Amico R., Frambolli I., Mele F., Filosto M., Vercelli L., Ruggiero L. (2013). Large scale genotype-phenotype analyses indicate that novel prognostic tools are required for families with facioscapulohumeral muscular dystrophy. Brain J. Neurol..

[3] Fecek C., Emmady P.D. (2021). Facioscapulohumeral Muscular Dystrophy. StatPearls.

[4] Ricci G., Ruggiero L., Vercelli L., Sera F., Nikolic A., Govi M., Mele F., Daolio J., Angelini C., Antonini G. (2016). A novel clinical tool to classify facioscapulohumeral muscular dystrophy phenotypes. J. Neurol..

[5] Vercelli L., Mele F., Ruggiero L., Sera F., Tripodi S., Ricci G., Vallarola A., Villa L., Govi M., Maranda L. (2021). A 5-year clinical follow-up study from the Italian National Registry for FSHD. J. Neurol..

[6] Lemmers R.J., Goeman J.J., van der Vliet P.J., van Nieuwenhuizen M.P., Balog J., Vos-Versteeg M., Camano P., Ramos Arroyo M.A., Jerico I., Rogers M.T. (2015). Inter-individual differences in CpG methylation at D4Z4 correlate with clinical variability in FSHD1 and FSHD2. Hum. Mol. Genet..

[7] Van den Boogaard M.L., Lemmers R., Balog J., Wohlgemuth M., Auranen M., Mitsuhashi S., van der Vliet P.J., Straasheijm K.R., van den Akker R.F.P., Kriek M. (2016). Mutations in DNMT3B Modify Epigenetic Repression of the D4Z4 Repeat and the Penetrance of Facioscapulohumeral Dystrophy. Am. J. Hum. Genet..

[8] Hamanaka K., Šikrová D., Mitsuhashi S., Masuda H., Sekiguchi Y., Sugiyama A., Shibuya K., Lemmers R., Goossens R., Ogawa M. (2020). Homozygous nonsense variant in LRIF1 associated with facioscapulohumeral muscular dystrophy. Neurology.

[9] Katz N.K., Hogan J., Delbango R., Cernik C., Tawil R., Statland J.M. (2021). Predictors of functional outcomes in patients with facioscapulohumeral muscular dystrophy. Brain J. Neurol..

[10] Wagner K.R. (2019). Facioscapulohumeral Muscular Dystrophies. Contin. Lifelong Learn. Neurol..

[11] Wijmenga C., Hewitt J.E., Sandkuijl L.A., Clark L.N., Wright T.J., Dauwerse H.G., Gruter A.M., Hofker M.H., Moerer P., Williamson R. (1992). Chromosome 4q DNA rearrangements associated with facioscapulohumeral muscular dystrophy. Nat. Genet..

[12] van Deutekom J.C., Wijmenga C., van Tienhoven E.A., Gruter A.M., Hewitt J.E., Padberg G.W., van Ommen G.J., Hofker M.H., Frants R.R. (1993). FSHD associated DNA rearrangements are due to deletions of integral copies of a 3.2 kb tandemly repeated unit. Hum. Mol. Genet..

[13] van Overveld P.G., Lemmers R.J., Deidda G., Sandkuijl L., Padberg G.W., Frants R.R., van der Maarel S.M. (2000). Interchromosomal repeat array interactions between chromosomes 4 and 10: A model for subtelomeric plasticity. Hum. Mol. Genet..

[14] Wohlgemuth M., Lemmers R.J., van der Kooi E.L., van der Wielen M.J., van Overveld P.G., Dauwerse H., Bakker E., Frants R.R., Padberg G.W., van der Maarel S.M. (2003). Possible phenotypic dosage effect in patients compound heterozygous for FSHD-sized 4q35 alleles. Neurology.

[15] DeSimone A.M., Leszyk J., Wagner K., Emerson C.P. (2019). Identification of the hyaluronic acid pathway as a therapeutic target for facioscapulohumeral muscular dystrophy. Sci. Adv..

[16] DeSimone A.M., Pakula A., Lek A., Emerson C.P. (2017). Facioscapulohumeral Muscular Dystrophy. Compr. Physiol..

[17] Wallace L.M., Garwick S.E., Mei W., Belayew A., Coppee F., Ladner K.J., Guttridge D., Yang J., Harper S.Q. (2011). *DUX4*, a candidate gene for facioscapulohumeral muscular dystrophy, causes p53-dependent myopathy in vivo. Ann. Neurol..

[18] Bosnakovski D., Gearhart M.D., Toso E.A., Recht O.O., Cucak A., Jain A.K., Barton M.C., Kyba M. (2017). p53-independent DUX4 pathology in cell and animal models of facioscapulohumeral muscular dystrophy. Dis. Models Mech..

[19] Dmitriev P., Bou Saada Y., Dib C., Ansseau E., Barat A., Hamade A., Dessen P., Robert T., Lazar V., Louzada R.A.N. (2016). DUX4-induced constitutive DNA damage and oxidative stress contribute to aberrant differentiation of myoblasts from FSHD patients. Free Radic. Biol. Med..

[20] Bosnakovski D., Xu Z., Gang E.J., Galindo C.L., Liu M., Simsek T., Garner H.R., Agha-Mohammadi S., Tassin A., Coppée F. (2008). An isogenetic myoblast expression screen identifies DUX4-mediated FSHD-associated molecular pathologies. EMBO J..

[21] Celegato B., Capitanio D., Pescatori M., Romualdi C., Pacchioni B., Cagnin S., Viganò A., Colantoni L., Begum S., Ricci E. (2006). Parallel protein and transcript profiles of FSHD patient muscles correlate to the D4Z4 arrangement and reveal a common impairment of slow to fast fibre differentiation and a general deregulation of MyoD-dependent genes. Proteomics.

[22] Haynes P., Kernan K., Zhou S.L., Miller D.G. (2017). Expression patterns of FSHD-causing DUX4 and myogenic transcription factors PAX3 and PAX7 are spatially distinct in differentiating human stem cell cultures. Skelet. Muscle.

[23] Dmitriev P., Kiseleva E., Kharchenko O., Ivashkin E., Pichugin A., Dessen P., Robert T., Coppée F., Belayew A., Carnac G. (2016). Dux4 controls migration of mesenchymal stem cells through the Cxcr4-Sdf1 axis. Oncotarget.

[24] Rickard A.M., Petek L.M., Miller D.G. (2015). Endogenous DUX4 expression in FSHD myotubes is sufficient to cause cell death and disrupts RNA splicing and cell migration pathways. Hum. Mol. Genet..

[25] Homma S., Beermann M.L., Boyce F.M., Miller J.B. (2015). Expression of FSHD-related DUX4-FL alters proteostasis and induces TDP-43 aggregation. Ann. Clin. Transl. Neurol..

[26] Shadle S.C., Zhong J.W., Campbell A.E., Conerly M.L., Jagannathan S., Wong C.J., Morello T.D., van der Maarel S.M., Tapscott S.J. (2017). DUX4-induced dsRNA and MYC mRNA stabilization activate apoptotic pathways in human cell models of facioscapulohumeral dystrophy. PLoS Genet..

[27] ReDUX4 Trial with Losmapimod in Facioscapulohumeral Muscular Dystrophy (FSHD) Demonstrating Slowed Disease Progression and Improved Function. https://ir.fulcrumtx.com/node/8066/pdf.

[28] de Valle K., Dobson F., Woodcock I., Carroll K., Ryan M.M., Heatwole C., Eichinger K., McGinley J.L. (2021). Reliability and validity of the FSHD-composite outcome measure in childhood facioscapulohumeral dystrophy. Neuromuscul. Disord. NMD.

[29] Compston A. (2010). Aids to the investigation of peripheral nerve injuries. Medical Research Council: Nerve Injuries Research Committee. His Majesty’s Stationery Office: 1942; pp. 48 (iii) and 74 figures and 7 diagrams; with aids to the examination of the peripheral nervous system. By Michael O’Brien for the Guarantors of Brain. Saunders Elsevier: 2010; pp. [8] 64 and 94 Figures. Brain J. Neurol..

[30] John J. (1984). Grading of muscle power: Comparison of MRC and analogue scales by physiotherapists. Medical Research Council. Int. J. Rehabil. Res..

[31] Andres P.L., Skerry L.M., Thornell B., Portney L.G., Finison L.J., Munsat T.L. (1996). A comparison of three measures of disease progression in ALS. J. Neurol. Sci..

[32] Brinkmann J.R. (1994). Comparison of a hand-held and fixed dynamometer in measuring strength of patients with neuromuscular disease. J. Orthop. Sports Phys. Ther..

[33] Paganoni S., Cudkowicz M., Berry J.D. (2014). Outcome measures in amyotrophic lateral sclerosis clinical trials. Clin. Investig..

[34] Visser J., Mans E., de Visser M., van den Berg-Vos R.M., Franssen H., de Jong J.M., van den Berg L.H., Wokke J.H., de Haan R.J. (2003). Comparison of maximal voluntary isometric contraction and hand-held dynamometry in measuring muscle strength of patients with progressive lower motor neuron syndrome. Neuromuscul. Disord. NMD.

[35] Baschung Pfister P., de Bruin E.D., Sterkele I., Maurer B., de Bie R.A., Knols R.H. (2018). Manual muscle testing and hand-held dynamometry in people with inflammatory myopathy: An intra- and interrater reliability and validity study. PLoS ONE.

[36] Hoagland R.J., Mendoza M., Armon C., Barohn R.J., Bryan W.W., Goodpasture J.C., Miller R.G., Parry G.J., Petajan J.H., Ross M.A. (1997). Reliability of maximal voluntary isometric contraction testing in a multicenter study of patients with amyotrophic lateral sclerosis. Muscle Nerve.

[37] Personius K.E., Pandya S., King W.M., Tawil R., McDermott M.P., The FSH DY Group (1994). Facioscapulohumeral dystrophy natural history study: Standardization of testing procedures and reliability of measurements. Phys. Ther..

[38] Tawil R., McDermott M.P., Mendell J.R., Kissel J., Griggs R.C., FSH-DY Group (1994). Facioscapulohumeral muscular dystrophy (FSHD): Design of natural history study and results of baseline testing. Neurology.

[39] The FSH-DY Group (1997). A prospective, quantitative study of the natural history of facioscapulohumeral muscular dystrophy (FSHD): Implications for therapeutic trials. Neurology.

[40] Gershman A., Chiang K., Do M., Abbink E., Harbers V., Audebert C., Campana-Salort E., Monforte M., Iyadurai S., Carey L. (2016). P.263—A randomized, double-blinded, placebo-controlled, multiple ascending dose study to evaluate the safety, tolerability, pharmacokinetics, immunogenicity, and biological activity of ATYR1940 in adult patients with facioscapulohumeral muscular dystrophy (FSHD). Neuromuscul. Disord..

[41] Walter M.C., Lochmüller H., Reilich P., Klopstock T., Huber R., Hartard M., Hennig M., Pongratz D., Müller-Felber W. (2000). Creatine monohydrate in muscular dystrophies: A double-blind, placebo-controlled clinical study. Neurology.

[42] Tawil R., McDermott M.P., Pandya S., King W., Kissel J., Mendell J.R., Griggs R.C., FSH-DY Group (1997). A pilot trial of prednisone in facioscapulohumeral muscular dystrophy. Neurology.

[43] Passerieux E., Hayot M., Jaussent A., Carnac G., Gouzi F., Pillard F., Picot M.C., Böcker K., Hugon G., Pincemail J. (2015). Effects of vitamin C, vitamin E, zinc gluconate, and selenomethionine supplementation on muscle function and oxidative stress biomarkers in patients with facioscapulohumeral dystrophy: A double-blind randomized controlled clinical trial. Free Radic. Biol. Med..

[44] ClinicalTrials.gov A Phase 2 Randomized, Double-Blind, Placebo-Controlled Study of ACE-083 in Patients with Facioscapulohumeral Muscular Dystrophy (NCT02927080). NCT02927080.

[45] ClinicalTrials.gov Study of Testosterone and rHGH in FSHD (STARFISH) (NCT03123913). NCT03123913.

[46] Statland J.M., McDermott M.P., Heatwole C., Martens W.B., Pandya S., van der Kooi E.L., Kissel J.T., Wagner K.R., Tawil R. (2013). Reevaluating measures of disease progression in facioscapulohumeral muscular dystrophy. Neuromuscul. Disord. NMD.

[47] Kissel J.T., McDermott M.P., Mendell J.R., King W.M., Pandya S., Griggs R.C., Tawil R. (2001). Randomized, double-blind, placebo-controlled trial of albuterol in facioscapulohumeral dystrophy. Neurology.

[48] van der Kooi E.L., Vogels O.J., van Asseldonk R.J., Lindeman E., Hendriks J.C., Wohlgemuth M., van der Maarel S.M., Padberg G.W. (2004). Strength training and albuterol in facioscapulohumeral muscular dystrophy. Neurology.

[49] Wagner K.R., Fleckenstein J.L., Amato A.A., Barohn R.J., Bushby K., Escolar D.M., Flanigan K.M., Pestronk A., Tawil R., Wolfe G.I. (2008). A phase I/IItrial of MYO-029 in adult subjects with muscular dystrophy. Ann. Neurol..

[50] Wohlgemuth M., van der Kooi E.L., van Kesteren R.G., van der Maarel S.M., Padberg G.W. (2004). Ventilatory support in facioscapulohumeral muscular dystrophy. Neurology.

[51] Kilmer D.D., Abresch R.T., McCrory M.A., Carter G.T., Fowler W.M., Johnson E.R., McDonald C.M. (1995). Profiles of neuromuscular diseases. Facioscapulohumeral muscular dystrophy. Am. J. Phys. Med. Rehabil..

[52] Tawil R., Kissel J.T., Heatwole C., Pandya S., Gronseth G., Benatar M. (2015). Evidence-based guideline summary: Evaluation, diagnosis, and management of facioscapulohumeral muscular dystrophy: Report of the Guideline Development, Dissemination, and Implementation Subcommittee of the American Academy of Neurology and the Practice Issues Review Panel of the American Association of Neuromuscular & Electrodiagnostic Medicine. Neurology.

[53] Wohlgemuth M., Horlings C.G.C., van der Kooi E.L., Gilhuis H.J., Hendriks J.C.M., van der Maarel S.M., van Engelen B.G.M., Heijdra Y.F., Padberg G.W. (2017). Respiratory function in facioscapulohumeral muscular dystrophy 1. Neuromuscul. Disord. NMD.

[54] Santos D.B., Boussaid G., Stojkovic T., Orlikowski D., Letilly N., Behin A., Butel S., Lofaso F., Prigent H. (2015). Respiratory muscle dysfunction in facioscapulohumeral muscular dystrophy. Neuromuscul. Disord. NMD.

[55] Moreira S., Wood L., Smith D., Marini-Bettolo C., Guglieri M., McMacken G., Bailey G., Mayhew A., Muni-Lofra R., Eglon G. (2017). Respiratory involvement in ambulant and non-ambulant patients with facioscapulohumeral muscular dystrophy. J. Neurol..

[56] Stübgen J.P., Schultz C. (2009). Lung and respiratory muscle function in facioscapulohumeral muscular dystrophy. Muscle Nerve.

[57] Henke C., Spiesshoefer J., Kabitz H.J., Herkenrath S., Randerath W., Brix T., Görlich D., Young P., Boentert M. (2019). Respiratory muscle weakness in facioscapulohumeral muscular dystrophy. Muscle Nerve.

[58] D’Angelo M.G., Romei M., Lo Mauro A., Marchi E., Gandossini S., Bonato S., Comi G.P., Magri F., Turconi A.C., Pedotti A. (2011). Respiratory pattern in an adult population of dystrophic patients. J. Neurol. Sci..

[59] (2002). ATS statement: Guidelines for the six-minute walk test. Am. J. Respir. Crit. Care Med..

[60] Mendell J.R., Goemans N., Lowes L.P., Alfano L.N., Berry K., Shao J., Kaye E.M., Mercuri E. (2016). Longitudinal effect of eteplirsen versus historical control on ambulation in Duchenne muscular dystrophy. Ann. Neurol..

[61] Eichinger K., Heatwole C., Heininger S., Stinson N., Matichak Stock C., Grosmann C., Wagner K.R., Tawil R., Statland J.M. (2017). Validity of the 6 min walk test in facioscapulohumeral muscular dystrophy. Muscle Nerve.

[62] Casanova C., Celli B.R., Barria P., Casas A., Cote C., de Torres J.P., Jardim J., Lopez M.V., Marin J.M., Montes de Oca M. (2011). The 6-min walk distance in healthy subjects: Reference standards from seven countries. Eur. Respir. J..

[63] Payan C.A., Hogrel J.Y., Hammouda E.H., Lacomblez L., Ollivier G., Doppler V., Eymard B., Attarian S., Pouget J., Desnuelle C. (2009). Periodic salbutamol in facioscapulohumeral muscular dystrophy: A randomized controlled trial. Arch. Phys. Med. Rehabil..

[64] Sitzia C., Meregalli M., Belicchi M., Farini A., Arosio M., Bestetti D., Villa C., Valenti L., Brambilla P., Torrente Y. (2019). Preliminary Evidences of Safety and Efficacy of Flavonoids- and Omega 3-Based Compound for Muscular Dystrophies Treatment: A Randomized Double-Blind Placebo Controlled Pilot Clinical Trial. Front. Neurol..

[65] van der Kooi E.L., de Greef J.C., Wohlgemuth M., Frants R.R., van Asseldonk R.J., Blom H.J., van Engelen B.G., van der Maarel S.M., Padberg G.W. (2006). No effect of folic acid and methionine supplementation on D4Z4 methylation in patients with facioscapulohumeral muscular dystrophy. Neuromuscul. Disord. NMD.

[66] Statland J., Amato A., Bravver E., Campbell C., Elman L., Johnson N., Joyce N., Karam C., Kissel J., Korngut L. (2018). Preliminary Results from a Phase 2 Study to Evaluate ACE-083, a Local Muscle Therapeutic, in Patients with Facioscapulohumeral Muscular Dystrophy (S38.001). Neurology.

[67] Haas B., Clarke E., Elver L., Gowman E., Mortimer E., Byrd E. (2019). The reliability and validity of the L-test in people with Parkinson’s disease. Physiotherapy.

[68] Bohannon R.W. (1997). Comfortable and maximum walking speed of adults aged 20–79 years: Reference values and determinants. Age Ageing.

[69] Moxley R.T. (1990). Functional testing. Muscle Nerve.

[70] Huisinga J., Bruetsch A., McCalley A., Currence M., Herbelin L., Jawdat O., Pasnoor M., Dimachkie M., Barohn R., Statland J. (2018). An instrumented timed up and go in facioscapulohumeral muscular dystrophy. Muscle Nerve.

[71] Statland J.M., Karanevich A., Bruetsch A., Huisinga J. (2019). A pilot study of the responsiveness of wireless motion analysis in facioscapulohumeral muscular dystrophy. Muscle Nerve.

[72] Lamperti C., Fabbri G., Vercelli L., D’Amico R., Frusciante R., Bonifazi E., Fiorillo C., Borsato C., Cao M., Servida M. (2010). A standardized clinical evaluation of patients affected by facioscapulohumeral muscular dystrophy: The FSHD clinical score. Muscle Nerve.

[73] Statland J.M., Heatwole C., Eichinger K., Dilek N., Martens W.B., Tawil R. (2016). Electrical impedance myography in facioscapulohumeral muscular dystrophy. Muscle Nerve.

[74] Han J.J., Kurillo G., Abresch R.T., de Bie E., Nicorici A., Bajcsy R. (2015). Reachable workspace in facioscapulohumeral muscular dystrophy (FSHD) by Kinect. Muscle Nerve.

[75] Han J.J., De Bie E., Nicorici A., Abresch R.T., Bajcsy R., Kurillo G. (2015). Reachable workspace reflects dynamometer-measured upper extremity strength in facioscapulohumeral muscular dystrophy. Muscle Nerve.

[76] Hatch M.N., Kurillo G., Chan V., Han J.J. (2021). Motion sensor-acquired reachable workspace correlates with patient-reported upper extremity activities of daily living (ADL) function in facioscapulohumeral dystrophy. Muscle Nerve.

[77] Eichinger K., Heatwole C., Iyadurai S., King W., Baker L., Heininger S., Bartlett A., Dilek N., Martens W.B., McDermott M. (2018). Facioscapulohumeral muscular dystrophy functional composite outcome measure. Muscle Nerve.

[78] Clinicaltrials.gov Clinical Trial Readiness to Solve Barriers to Drug Development in FSHD (NCT03458832). NCT03458832.

[79] Ricci E., Galluzzi G., Deidda G., Cacurri S., Colantoni L., Merico B., Piazzo N., Servidei S., Vigneti E., Pasceri V. (1999). Progress in the molecular diagnosis of facioscapulohumeral muscular dystrophy and correlation between the number of KpnI repeats at the 4q35 locus and clinical phenotype. Ann. Neurol..

[80] van Overveld P.G., Enthoven L., Ricci E., Rossi M., Felicetti L., Jeanpierre M., Winokur S.T., Frants R.R., Padberg G.W., van der Maarel S.M. (2005). Variable hypomethylation of D4Z4 in facioscapulohumeral muscular dystrophy. Ann. Neurol..

[81] Mul K., Vincenten S.C.C., Voermans N.C., Lemmers R.J.L.F., van der Vliet P.J., van der Maarel S.M., Padberg G.W., Horlings C.G.C., van Engelen B.G.M. (2017). Adding quantitative muscle MRI to the FSHD clinical trial toolbox. Neurology.

[82] Bérard C., Payan C., Hodgkinson I., Fermanian J. (2005). A motor function measure for neuromuscular diseases. Construction and validation study. Neuromuscul. Disord. NMD.

[83] Bérard C., Payan C., Fermanian J., Girardot F. (2006). A motor function measurement scale for neuromuscular diseases-description and validation study. Rev. Neurol..

[84] Fischmann A., Gloor M., Fasler S., Haas T., Rodoni Wetzel R., Bieri O., Wetzel S., Heinimann K., Scheffler K., Fischer D. (2011). Muscular involvement assessed by MRI correlates to motor function measurement values in oculopharyngeal muscular dystrophy. J. Neurol..

[85] de Lattre C., Payan C., Vuillerot C., Rippert P., de Castro D., Bérard C., Poirot I. (2013). Motor function measure: Validation of a short form for young children with neuromuscular diseases. Arch. Phys. Med. Rehabil..

[86] van Eijk R.P.A., Beelen A., Kruitwagen E.T., Murray D., Radakovic R., Hobson E., Knox L., Helleman J., Burke T., Rubio Pérez M. (2021). A Road Map for Remote Digital Health Technology for Motor Neuron Disease. J. Med. Internet Res..

[87] Berry J.D., Paganoni S., Carlson K., Burke K., Weber H., Staples P., Salinas J., Chan J., Green J.R., Connaghan K. (2019). Design and results of a smartphone-based digital phenotyping study to quantify ALS progression. Ann. Clin. Transl. Neurol..

[88] Beukenhorst A.L., Collins E., Burke K.M., Rahman S.M., Clapp M., Konanki S.C., Paganoni S., Miller T.M., Chan J., Onnela J.P. (2021). Smartphone data during the COVID-19 pandemic can quantify behavioral changes in people with ALS. Muscle Nerve.

[89] Gidaro T., Gasnier E., Denis S., Lilien C., Grelet M., Rigaud A., Moraux A., Dorveaux E., Vissiere D., Servais L. (2016). Assessment of lower limbs in FSHD: The ActiMyo as a new outcome for home-monitoring. Neuromuscul. Disord. NMD.

[90] Gidaro T., Moraux A., Grelet M., Gasnier E., Villeret M., Annoussamy M., Vissing J., Attarian S., Mozaffar T., Iyadurai S. (2017). ActiMyo home monitoring in adult patients with limb girdle muscular dystrophy type 2B and facioscapulohumeral muscular dystrophy in study ATYR 1940-C-004. Neuromuscul. Disord. NMD.

[91] Gidaro T., Gasnier E., Annoussamy M., Vissing J., Attarian S., Mozaffar T., Iyadurai S., Wagner K.R., Vissière D., Walker G. (2021). Home-based gait analysis as an exploratory endpoint during a multicenter phase 1 trial in limb girdle muscular dystrophy type R2 and facioscapulohumeral muscular dystrophy. Muscle Nerve.

[92] Zhuparris A. Exploratory study to biotype patients with facioscapulohumeral muscular dystrophy (FSHD) and controls using digital technologies: Preliminary results. Proceedings of the 2020 MDA Clinical and Scientific Conference.

[93] Mul K., Hamadeh T., Horlings C.G.C., Tawil R., Statland J.M., Sacconi S., Corbett A.J., Voermans N.C., Faber C.G., van Engelen B.G.M. (2021). The facioscapulohumeral muscular dystrophy Rasch-built overall disability scale (FSHD-RODS). Eur. J. Neurol..

[94] Wright B.D., Linacre J.M. (1989). Observations are always ordinal; measurements, however, must be interval. Arch. Phys. Med. Rehabil..

[95] Johnson N.E., Quinn C., Eastwood E., Tawil R., Heatwole C.R. (2012). Patient-identified disease burden in facioscapulohumeral muscular dystrophy. Muscle Nerve.

[96] Tawil R., Padberg G.W., Shaw D.W., van der Maarel S.M., Tapscott S.J. (2016). Clinical trial preparedness in facioscapulohumeral muscular dystrophy: Clinical, tissue, and imaging outcome measures 29–30 May 2015, Rochester, New York. Neuromuscul. Disord. NMD.

[97] LoRusso S., Johnson N.E., McDermott M.P., Eichinger K., Butterfield R.J., Carraro E., Higgs K., Lewis L., Mul K., Sacconi S. (2019). Clinical trial readiness to solve barriers to drug development in FSHD (ReSolve): Protocol of a large, international, multi-center prospective study. BMC Neurol..

[98] Vandervelde L., Van den Bergh P.Y., Goemans N., Thonnard J.L. (2007). ACTIVLIM: A Rasch-built measure of activity limitations in children and adults with neuromuscular disorders. Neuromuscul. Disord. NMD.

[99] Batcho C.S., Van den Bergh P.Y., Van Damme P., Roy A.J., Thonnard J.L., Penta M. (2016). How robust is ACTIVLIM for the follow-up of activity limitations in patients with neuromuscular diseases?. Neuromuscul. Disord. NMD.

[100] De Wel B., Goosens V., Sobota A., Van Camp E., Geukens E., Van Kerschaver G., Jagut M., Claes K., Claeys K.G. (2021). Nusinersen treatment significantly improves hand grip strength, hand motor function and MRC sum scores in adult patients with spinal muscular atrophy types 3 and 4. J. Neurol..

[101] Vanherpe P., Fieuws S., D’Hondt A., Bleyenheuft C., Demaerel P., De Bleecker J., Van den Bergh P., Baets J., Remiche G., Verhoeven K. (2020). Late-onset Pompe disease (LOPD) in Belgium: Clinical characteristics and outcome measures. Orphanet J. Rare Dis..

[102] Nicolau S., Naddaf E. (2020). Muscle MRI for Neuromuscular Disorders. Pract. Neurol..

[103] Leung D.G., Carrino J.A., Wagner K.R., Jacobs M.A. (2015). Whole-body magnetic resonance imaging evaluation of facioscapulohumeral muscular dystrophy. Muscle Nerve.

[104] Gerevini S., Scarlato M., Maggi L., Cava M., Caliendo G., Pasanisi B., Falini A., Previtali S.C., Morandi L. (2016). Muscle MRI findings in facioscapulohumeral muscular dystrophy. Eur. Radiol..

[105] Jordan B., Eger K., Koesling S., Zierz S. (2011). Camptocormia phenotype of FSHD: A clinical and MRI study on six patients. J. Neurol..

[106] Regula J.U., Jestaedt L., Jende F., Bartsch A., Meinck H.M., Weber M.A. (2016). Clinical Muscle Testing Compared with Whole-Body Magnetic Resonance Imaging in Facio-scapulo-humeral Muscular Dystrophy. Clin. Neuroradiol..

[107] Olsen D.B., Gideon P., Jeppesen T.D., Vissing J. (2006). Leg muscle involvement in facioscapulohumeral muscular dystrophy assessed by MRI. J. Neurol..

[108] Wang L.H., Shaw D.W.W., Faino A., Budech C.B., Lewis L.M., Statland J., Eichinger K., Tapscott S.J., Tawil R.N., Friedman S.D. (2021). Longitudinal study of MRI and functional outcome measures in facioscapulohumeral muscular dystrophy. BMC Musculoskelet Disord..

[109] Monforte M., Laschena F., Ottaviani P., Bagnato M.R., Pichiecchio A., Tasca G., Ricci E. (2019). Tracking muscle wasting and disease activity in facioscapulohumeral muscular dystrophy by qualitative longitudinal imaging. J. Cachexia Sarcopenia Muscle.

[110] Tasca G., Pescatori M., Monforte M., Mirabella M., Iannaccone E., Frusciante R., Cubeddu T., Laschena F., Ottaviani P., Ricci E. (2012). Different molecular signatures in magnetic resonance imaging-staged facioscapulohumeral muscular dystrophy muscles. PLoS ONE.

[111] Ferguson M.R., Poliachik S.L., Budech C.B., Gove N.E., Carter G.T., Wang L.H., Miller D.G., Shaw D.W.W., Friedman S.D. (2018). MRI change metrics of facioscapulohumeral muscular dystrophy: Stir and T1. Muscle Nerve.

[112] Andersen G., Dahlqvist J.R., Vissing C.R., Heje K., Thomsen C., Vissing J. (2017). MRI as outcome measure in facioscapulohumeral muscular dystrophy: 1-year follow-up of 45 patients. J. Neurol..

[113] Fatehi F., Salort-Campana E., Le Troter A., Lareau-Trudel E., Bydder M., Fouré A., Guye M., Bendahan D., Attarian S. (2017). Long-term follow-up of MRI changes in thigh muscles of patients with Facioscapulohumeral dystrophy: A quantitative study. PLoS ONE.

[114] Dahlqvist J.R., Poulsen N.S., Østergaard S.T., Fornander F., de Stricker Borch J., Danielsen E.R., Thomsen C., Vissing J. (2020). Evaluation of inflammatory lesions over 2 years in facioscapulohumeral muscular dystrophy. Neurology.

[115] Friedman S.D., Poliachik S.L., Carter G.T., Budech C.B., Bird T.D., Shaw D.W. (2012). The magnetic resonance imaging spectrum of facioscapulohumeral muscular dystrophy. Muscle Nerve.

[116] Wang L.H., Friedman S.D., Shaw D., Snider L., Wong C.-J., Budech C.B., Poliachik S.L., Gove N.E., Lewis L.M., Campbell A.E. (2019). MRI-informed muscle biopsies correlate MRI with pathology and DUX4 target gene expression in FSHD. Hum. Mol. Genet..

[117] Frisullo G., Frusciante R., Nociti V., Tasca G., Renna R., Iorio R., Patanella A.K., Iannaccone E., Marti A., Rossi M. (2011). CD8(+) T cells in facioscapulohumeral muscular dystrophy patients with inflammatory features at muscle MRI. J. Clin. Immunol..

[118] Lassche S., Küsters B., Heerschap A., Schyns M.V.P., Ottenheijm C.A.C., Voermans N.C., van Engelen B.G.M. (2020). Correlation between Quantitative MRI and Muscle Histopathology in Muscle Biopsies from Healthy Controls and Patients with IBM, FSHD and OPMD. J. Neuromuscul. Dis..

[119] Wijntjes J., van Alfen N. (2021). Muscle ultrasound: Present state and future opportunities. Muscle Nerve.

[120] Jansen M., van Alfen N., Nijhuis van der Sanden M.W., van Dijk J.P., Pillen S., de Groot I.J. (2012). Quantitative muscle ultrasound is a promising longitudinal follow-up tool in Duchenne muscular dystrophy. Neuromuscul. Disord. NMD.

[121] Zaidman C.M., Wu J.S., Kapur K., Pasternak A., Madabusi L., Yim S., Pacheck A., Szelag H., Harrington T., Darras B.T. (2017). Quantitative muscle ultrasound detects disease progression in Duchenne muscular dystrophy. Ann. Neurol..

[122] Janssen B.H., Pillen S., Voet N.B., Heerschap A., van Engelen B.G., van Alfen N. (2014). Quantitative muscle ultrasound versus quantitative magnetic resonance imaging in facioscapulohumeral dystrophy. Muscle Nerve.

[123] Gijsbertse K., Goselink R., Lassche S., Nillesen M., Sprengers A., Verdonschot N., van Alfen N., de Korte C. (2017). Ultrasound Imaging of Muscle Contraction of the Tibialis Anterior in Patients with Facioscapulohumeral Dystrophy. Ultrasound Med. Biol..

[124] Mul K., Horlings C.G.C., Vincenten S.C.C., Voermans N.C., van Engelen B.G.M., van Alfen N. (2018). Quantitative muscle MRI and ultrasound for facioscapulohumeral muscular dystrophy: Complementary imaging biomarkers. J. Neurol..

[125] Veltsista D., Chroni E. (2021). Ultrasound pattern of anterolateral leg muscles in facioscapulohumeral muscular dystrophy. Acta Neurol. Scand..

[126] Goselink R.J.M., Schreuder T.H.A., Mul K., Voermans N.C., Erasmus C.E., van Engelen B.G.M., van Alfen N. (2020). Muscle ultrasound is a responsive biomarker in facioscapulohumeral dystrophy. Neurology.

[127] Rutkove S.B. (2009). Electrical impedance myography: Background, current state, and future directions. Muscle Nerve.

[128] Rutkove S.B., Caress J.B., Cartwright M.S., Burns T.M., Warder J., David W.S., Goyal N., Maragakis N.J., Clawson L., Benatar M. (2012). Electrical impedance myography as a biomarker to assess ALS progression. Amyotroph. Lateral Scler..

[129] Zaidman C.M., Wang L.L., Connolly A.M., Florence J., Wong B.L., Parsons J.A., Apkon S., Goyal N., Williams E., Escolar D. (2015). Electrical impedance myography in Duchenne muscular dystrophy and healthy controls: A multicenter study of reliability and validity. Muscle Nerve.

[130] Rutkove S.B., Shefner J.M., Gregas M., Butler H., Caracciolo J., Lin C., Fogerson P.M., Mongiovi P., Darras B.T. (2010). Characterizing spinal muscular atrophy with electrical impedance myography. Muscle Nerve.

[131] Hamel J., Lee P., Glenn M.D., Burka T., Choi I.Y., Friedman S.D., Shaw D.W.W., McCalley A., Herbelin L., Dimachkie M.M. (2020). Magnetic resonance imaging correlates with electrical impedance myography in facioscapulohumeral muscular dystrophy. Muscle Nerve.

[132] Mul K., Heatwole C., Eichinger K., Dilek N., Martens W.B., Van Engelen B.G.M., Tawil R., Statland J.M. (2018). Electrical impedance myography in facioscapulohumeral muscular dystrophy: A 1-year follow-up study. Muscle Nerve.

[133] Arahata K., Ishihara T., Fukunaga H., Orimo S., Lee J.H., Goto K., Nonaka I. (1995). Inflammatory response in facioscapulohumeral muscular dystrophy (FSHD): Immunocytochemical and genetic analyses. Muscle Nerve.

[134] Felisaz P.F., Colelli G., Ballante E., Solazzo F., Paoletti M., Germani G., Santini F., Deligianni X., Bergsland N., Monforte M. (2021). Texture analysis and machine learning to predict water T2 and fat fraction from non-quantitative MRI of thigh muscles in Facioscapulohumeral muscular dystrophy. Eur. J. Radiol..

[135] Banerji C.R.S., Zammit P.S. (2021). Pathomechanisms and biomarkers in facioscapulohumeral muscular dystrophy: Roles of DUX4 and PAX7. EMBO Mol. Med..

[136] Dixit M., Ansseau E., Tassin A., Winokur S., Shi R., Qian H., Sauvage S., Mattéotti C., van Acker A.M., Leo O. (2007). DUX4, a candidate gene of facioscapulohumeral muscular dystrophy, encodes a transcriptional activator of PITX1. Proc. Natl. Acad. Sci. USA.

[137] Vanderplanck C., Ansseau E., Charron S., Stricwant N., Tassin A., Laoudj-Chenivesse D., Wilton S.D., Coppée F., Belayew A. (2011). The FSHD atrophic myotube phenotype is caused by DUX4 expression. PLoS ONE.

[138] Broucqsault N., Morere J., Gaillard M.C., Dumonceaux J., Torrents J., Salort-Campana E., Maues De Paula A., Bartoli M., Fernandez C., Chesnais A.L. (2013). Dysregulation of 4q35- and muscle-specific genes in fetuses with a short D4Z4 array linked to facio-scapulo-humeral dystrophy. Hum. Mol. Genet..

[139] van den Heuvel A., Mahfouz A., Kloet S.L., Balog J., van Engelen B.G.M., Tawil R., Tapscott S.J., van der Maarel S.M. (2019). Single-cell RNA sequencing in facioscapulohumeral muscular dystrophy disease etiology and development. Hum. Mol. Genet..

[140] Jiang S., Williams K., Kong X., Zeng W., Nguyen N.V., Ma X., Tawil R., Yokomori K., Mortazavi A. (2020). Single-nucleus RNA-seq identifies divergent populations of FSHD2 myotube nuclei. PLoS Genet..

[141] Yao Z., Snider L., Balog J., Lemmers R.J.L.F., Van Der Maarel S.M., Tawil R., Tapscott S.J. (2014). DUX4-induced gene expression is the major molecular signature in FSHD skeletal muscle. Hum. Mol. Genet..

[142] Tassin A., Laoudj-Chenivesse D., Vanderplanck C., Barro M., Charron S., Ansseau E., Chen Y.W., Mercier J., Coppée F., Belayew A. (2013). DUX4 expression in FSHD muscle cells: How could such a rare protein cause a myopathy?. J. Cell. Mol. Med..

[143] Snider L., Geng L.N., Lemmers R.J., Kyba M., Ware C.B., Nelson A.M., Tawil R., Filippova G.N., van der Maarel S.M., Tapscott S.J. (2010). Facioscapulohumeral dystrophy: Incomplete suppression of a retrotransposed gene. PLoS Genet..

[144] Banerji C.R., Knopp P., Moyle L.A., Severini S., Orrell R.W., Teschendorff A.E., Zammit P.S. (2015). β-Catenin is central to DUX4-driven network rewiring in facioscapulohumeral muscular dystrophy. J. R. Soc. Interface.

[145] Statland J., Donlin-Smith C.M., Tapscott S.J., van der Maarel S., Tawil R. (2014). Multiplex Screen of Serum Biomarkers in Facioscapulohumeral Muscular Dystrophy. J. Neuromuscul. Dis..

[146] Petek L.M., Rickard A.M., Budech C., Poliachik S.L., Shaw D., Ferguson M.R., Tawil R., Friedman S.D., Miller D.G. (2016). A cross sectional study of two independent cohorts identifies serum biomarkers for facioscapulohumeral muscular dystrophy (FSHD). Neuromuscul. Disord. NMD.

[147] Gros M., Nunes A.M., Daoudlarian D., Pini J., Martinuzzi E., Barbosa S., Ramirez M., Puma A., Villa L., Cavalli M. (2021). Identification of Serum Interleukin 6 Levels as a Disease Severity Biomarker in Facioscapulohumeral Muscular Dystrophy. J. Neuromuscul. Dis..

[148] Tasca G., Monforte M., Corbi M., Granata G., Lucchetti D., Sgambato A., Ricci E. (2018). Muscle Microdialysis to Investigate Inflammatory Biomarkers in Facioscapulohumeral Muscular Dystrophy. Mol. Neurobiol..

[149] Greco A., Straasheijm K.R., Mul K., van den Heuvel A., van der Maarel S.M., Joosten L.A.B., van Engelen B.G.M., Pruijn G.J.M. (2021). Profiling Serum Antibodies against Muscle Antigens in Facioscapulohumeral Muscular Dystrophy Finds No Disease-Specific Autoantibodies. J. Neuromuscul. Dis..

[150] Nunes A.M., Ramirez M., Jones T.I., Jones P.L. (2021). Identification of candidate miRNA biomarkers for facioscapulohumeral muscular dystrophy using DUX4-based mouse models. Dis. Models Mech..

[151] Butz M., Koch M.C., Müller-Felber W., Lemmers R.J., van der Maarel S.M., Schreiber H. (2003). Facioscapulohumeral muscular dystrophy. Phenotype-genotype correlation in patients with borderline D4Z4 repeat numbers. J. Neurol..

[152] Salort-Campana E., Nguyen K., Bernard R., Jouve E., Solé G., Nadaj-Pakleza A., Niederhauser J., Charles E., Ollagnon E., Bouhour F. (2015). Low penetrance in facioscapulohumeral muscular dystrophy type 1 with large pathological D4Z4 alleles: A cross-sectional multicenter study. Orphanet J. Rare Dis..

[153] He J.J., Lin X.D., Lin F., Xu G.R., Xu L.Q., Hu W., Wang D.N., Lin H.X., Lin M.T., Wang N. (2018). Clinical and genetic features of patients with facial-sparing facioscapulohumeral muscular dystrophy. Eur. J. Neurol..

[154] Ruggiero L., Mele F., Manganelli F., Bruzzese D., Ricci G., Vercelli L., Govi M., Vallarola A., Tripodi S., Villa L. (2020). Phenotypic Variability among Patients with D4Z4 Reduced Allele Facioscapulohumeral Muscular Dystrophy. JAMA Netw. Open.

